# The prevalence of childhood bereavement in Scotland and its relationship with disadvantage: the significance of a public health approach to death, dying and bereavement

**DOI:** 10.1177/2632352420975043

**Published:** 2020-12-02

**Authors:** Sally Paul, Nina Vaswani

**Affiliations:** University of Strathclyde, Lord Hope Building, 141 St James Road, Glasgow G4 0LT, UK; Children and Young People’s Centre for Justice, University of Strathclyde, Glasgow, UK

**Keywords:** bereavement, childhood, death, disadvantage, dying, inequalities, palliative care, public health, socioeconomic status

## Abstract

**Background and Method:**

There is an absence of research on the prevalence of bereavement during early childhood and the relationship between childhood bereavement and socioeconomic status (SES) and this poses a challenge in both understanding and supporting children’s bereavement experiences. Using longitudinal data from the Growing Up in Scotland study, which tracks the lives of three nationally representative cohorts of children, this paper aimed to address these gaps in research. It specifically drew on data from Birth Cohort 1 to document the recorded bereavements of 2,815 children who completed all 8 sweeps of data collection, from age 10 months to 10 years.

**Findings:**

The study found that 50.8% of all children are bereaved of a parent, sibling, grandparent or other close family member by age 8 and this rises to 62% by age 10. The most common death experienced was that of a grandparent or other close relative. The study also found that children born into the lowest income households are at greater risk of being bereaved of a parent or sibling than those born into the highest income households.

**Discussion and Conclusion:**

Given the prevalence of childhood bereavement and its relationship with disadvantage, this paper argues that there is an important need to understand bereavement as a universal issue that is affected by the social conditions in which a child becomes bereaved, as well as an individual experience potentially requiring specialist support. This paper thus seeks to position childhood bereavement more firmly within the public health approach to palliative and bereavement care discourse and contends that doing so provides a unique and comprehensive opportunity to better understand and holistically respond to the experience of bereavement during childhood.

## Introduction

Bereavement during childhood is a common experience but one that is frequently associated with a range of immediate and long-term risks related to social, emotional and physical wellbeing. That children grieve is widely accepted and many children facing a bereavement will experience intense and confusing emotions that are associated with ‘normal’ grief, including sadness, anger and confusion.^1^ While the majority of children do not need professional services,^2^ there is a body of research which argues that bereavement can make children vulnerable to a variety of risks, including anxiety, depression, prolonged grief disorder, self-harm, suicide, underachievement at school, offending and unemployment.^2–7^ However, a significant issue in both understanding and responding to children’s bereavement experiences is that the data on the prevalence of bereavement is limited, particularly in relation to younger children. In Scotland, there are no prevalence studies but a recent survey of 185 children aged 11–17 estimated that 72% had experienced a bereavement.^8^ This reflects estimates across the United Kingdom more widely: in England, it is estimated that 78% of 11- to 16-year-olds have experienced the death of a family member or close friend.^2^ In Great Britain, it is estimated that 4–7% of children will experience the death of a parent by the age of 16.^5^ These figures, although substantial, are based on smaller studies or drawn from mortality data and it is likely that the actual number of children experiencing bereavement is much higher. Moreover, little is known about when bereavement experiences start to occur over the life course and this poses a challenge in fully understanding how children are, and can be, supported over time including what supports are helpful and when.

The lack of baseline data on the prevalence of childhood bereavement is further challenged by the predominant emphasis within current research on problematizing the experience of bereavement, rather than focus on preventive care that recognizes and promotes resilience in communities.^9^ Thus, research on childhood bereavement tends to focus on risks, vulnerabilities and psychological adjustment to loss as opposed to the strengths and deficits of the wider social context. This is significant given that research would suggest that people experiencing multiple disadvantages encounter bereavement and loss at a disproportionate rate.^10,11^ Moreover, higher family socioeconomic status (SES) is associated with better outcomes for children experiencing bereavement.^12^ This research study aimed to attend to this lack of baseline data by exploring the prevalence of bereavement in younger children in Scotland, exploring when bereavement experiences occur over the life course and the relationship between bereavement and SES. The findings suggest that the prevalence of bereavement in younger children is much higher than previous estimates and that children with a lower household SES were significantly more likely to experience the death of a parent or sibling. As such, this article seeks to reframe childhood bereavement as a universal issue that demands collective support and innovation, alongside specialist service provision, and in doing so position children more firmly within discourse related to public health approaches to death, dying and bereavement.

## Childhood bereavement and the relevance of public health approaches to death, dying and bereavement

How a child engages with or copes with their bereavement is mediated by a variety of factors that relate not only to the bereavement itself (such as attachment to and relationship with the deceased, type of death and so on) but also to the child’s development (emotional, cognitive and social) and wider relational, social, educational and cultural environment.^11,13–15^ For some children, appropriate and timely specialist bereavement support is essential and in affluent societies a range of bereavement services exist. Yet, while there is a limited but growing evidence base on the strengths and weaknesses of such support,^16,17^ the majority of children do not require specialist intervention^18^ and the indiscriminate use of bereavement services can be unhelpful.^16^ Children are more likely to seek support from family and friends^19^ and communication and support both within families and from the wider community have been found to play an important role in children’s coping.^20,21^ However, a lack of social support (from schools, religious organizations, neighbours and friendship networks) has been found to contribute to feelings of isolation,^22^ loneliness and social exclusion^23^ and some children report bullying^24^ and difficult friendships.^25,26^ Thus, the role of, and capacity within, a child’s wider social and cultural context to support bereavement experiences cannot be ignored.

The integral role that communities have in responding to, and supporting, death, dying and loss is a significant concern for palliative care. This concern recognizes that the dying or bereaved cannot be (and are not being) supported by professionals alone and that care for these groups involves a multifaceted approach. As such, the relevance of public health for palliative care is well recognized for the contribution it can make to meaningful end of life care;^27–29^ yet, limited attention has been given to grief and loss as a public health issue. The recent work of Auon, Rumbold and colleagues has played an important role in addressing this gap.^30–33^ They argue that the numerous adverse consequences that are associated with bereavement, those that transcend emotional, cognitive and physical functioning, coupled with the disruptions of social relationships, firmly position bereavement as a public health issue. This argument is based on ‘new’ public health approaches that move away from traditional biomedical approaches to public health to a broader understanding that recognizes the significance of individuals within the context of their environment.^34^ As such, Aoun and colleagues^35^ argue for a population-based model of bereavement support that identifies the integral role of community supports in addressing the social epidemiology of loss and grief. In doing so, they assert that family, friends and social networks are basic to all bereavement needs, with other (specialist) supports being additional for a minority of people who need them.^35^ While this public health discourse can be critiqued for potentially managing the deficits in bereavement service provision,^36^ it also recognizes that most people live with and through bereavement. As such, emphasis is given to the types of supports that scaffold these experiences to mobilize that which is absent from individuals’ personal and social networks.^33^

There is an absence of children in current debates about the relevance of public health approaches to bereavement, and this mirrors discussion on public health approaches to palliative care more broadly. However, the idea of bereavement as a public health issue is arguably not new to literature on childhood bereavement support. Support for children experiencing bereavement is informed by research on the sociology of childhood which places emphasis on understanding the lives of children based on their own experiences, meanings and interpretations.^37^ As such, childhood is not viewed as natural or universal but is shaped by environmental and social factors. Literature on childhood bereavement therefore places emphasis not only on the individual, such as in relation to age, gender, cognitive ability and so on, but also to the immediate and wider social context. Accordingly, there is a variety of literature and practice guidance that recognizes the significance of children being able to access informal support from within their existing communities, if and where possible, and specialist support when, and if, needed.^18,20,38^ Although this literature refers to the significance of a universal approach to bereavement, it does not specifically identify the relevance of public health, rather this is implicit within the different models offered. Furthermore, research focuses predominately on acute models of therapeutic intervention^18^ and as such the experiences of children who are known to professional services: less is known about the experiences of children who do not receive professional support and this presents an important gap in theoretical and practice knowledge. Explicitly applying public health to childhood bereavement potentially offers an opportunity to address this gap. As a population-based approach, public health is concerned with how bereavement affects all children^39^ and focus is therefore given to prevention and equity of care whereby harm reduction and early intervention are key principles.^34^ In Scotland, this focus aligns with current national public health priorities on positive early years and mental wellbeing.^40^ This article contends that through developing a better understanding of the prevalence of childhood bereavement in Scotland and the relationship with SES, childhood bereavement can be recognized as a majority experience that is influenced by the social conditions within which children become bereaved. Below, we present the research methods and findings, going on to discuss how these shape our understanding of childhood bereavement as a public health issue that necessarily calls for a wider recognition of, and response to, the broader economic, social and cultural experiences of children.

## Methods

Data were drawn from the Growing Up in Scotland (GUS) longitudinal study which tracks the lives of three nationally representative cohorts of around 14,000 children across Scotland. Through face-to-face computer-assisted interviews with parents or carers, GUS collects a range of information relating to the child’s health and development, family circumstances and experiences, education and social characteristics. GUS is funded by the Scottish Government and administered by ScotCen Social Research in collaboration with the University of Glasgow. Cohorts were sampled at random from universal Child Benefit records held by HMRC and more detailed information on sampling and methodology is available.^41^ Datasets are stored by the UK Data Service and are accessible upon application.

### Ethics

Full ethical approval for the GUS study was given by the Scotland ‘A’ Multi-Centre Research Ethics Committee (application reference: 04/M RE 1 0/59). To ensure both parents/carers and the cohort children themselves are well-informed of their rights in relation to the survey, extensive study materials are provided to participants both in advance and at the point of data collection. The exact nature of materials differs slightly at each sweep to reflect the specific content as well as the child’s age. Written consent for participation in the study was obtained from the main adult respondent at the study outset. Verbal consent from parents and cohort members is sought for participation in the interview ahead of each study sweep and for each data collection element within a sweep. Consent to link survey data to health and education administrative records was obtained in writing from the main adult respondent. Participants are given the opportunity to reaffirm or withdraw data linkage consent at key intervals.

### Sample

The data used in this study were Birth Cohort 1, which contains 5217 children born between June 2004 and May 2005. Nine sweeps of data have been collected on this cohort, annually from between the ages of 10 months and 6 years, and every 2 years thereafter, with Sweep 10 currently underway. This cohort was selected as it provides the longest time span of data from which to calculate the prevalence of childhood bereavement. At the time of writing, only data up to and including Sweep 8 were available for analysis in this study. This sweep focused on ‘stage’ rather than ‘age’, with interviews aimed at children entering their first term of Primary 6 and conducted over two school years in 2014 and 2015. Sweep 8 included 3150 children, representing 60% of the original sample.

Only those children who had participated in each of the eight sweeps, and therefore had a complete dataset, were included in this analysis (*n* = 2815), which represents 54% of the original sample at Sweep 1, and 89% of Sweep 8 participants. Within the unweighted sample, 1436 children were male (51%) and 1379 were female (49%). The mean (unweighted) age at the time of Sweep 8 was 10.18 years (SD = 0.30), with a range of 9.5–11.4 years. The majority of children were of a White ethnic background (*n* = 2693, 95.7%) and the mean (unweighted) Equivalised Household Income at Sweep 8 was £26,251.28 (SD = £12,405).

### Variables

Bereavement Status was derived from the Significant Life Events variable. This variable was introduced in Sweep 2 and was worded ‘Can I check, has [childname] experienced any of the following since [the date of last interview]?’ In Sweep 2, the variable covered 11 significant life events. Only three items ‘death of a parent or parent figure’, ‘death of a brother or sister’ and ‘death of a grandparent or other close relative’ related to bereavement and the responses to these formed the basis of the analysis. The wording of the question did not materially change in subsequent sweeps, although additional items were included. The only new item of relevance to this study was ‘death of a pet’: because this was introduced at Sweep 6, data from this item were not included in this study. Responses as to whether any of the three measured bereavements had occurred since the previous sweep were recorded dichotomously in the dataset as a ‘yes’ or ‘no’.

Socioeconomic status was derived from the variable Equivalised Annual Household Income at Sweep 1. In this variable, the household income is adjusted for family size and composition to produce a more comparable income across participants. In this adjustment, an adult couple without children is used as the benchmark. A single adult is assumed to attain a comparable standard of living to a couple without children at two-thirds of the income (i.e. an equivalence scale of 0.67). Each child aged below 14 in the household is afforded an equivalence scale of 0.2 and those aged 14–18 is afforded 0.33.^42^ For example, an adult couple with three children aged 3, 5 and 10 would have an equivalence scale of 1.6 (i.e. they are assumed to need 60% more income than a couple with no children to attain a comparable standard of living).

Additional variables used as proxy indicators of family SES or family disadvantage/stressors were Household tenure type at Sweep 1 in which data were collapsed into two possible responses: owner occupied or rented (the latter including private rented, social rented and ‘other’ housing); and parents/carers in receipt of Disability Living Allowance (DLA) at Sweep 1. DLA was a benefit available to individuals who met the eligibility requirements for care or mobility payments (or both) but was phased out from 2013 and replaced with the Personal Independence Payment. The criteria for the ‘care’ component of DLA included individuals who required help with everyday tasks such as cooking, washing or dressing. The mobility component was for individuals who experienced severe difficulties walking without assistance.^43^

### Analysis

Data were analysed using SPSS Statistics, version 25.^44^ Longitudinal weights for Sweep 8, as calculated by ScotCen, were applied to the data, which adjusts for unequal selection probabilities and bias in the sample, and allows for inferences to be made about the national population. No imputations were carried out for missing data. Data from each individual sweep were linked to calculate the lifetime prevalence of childhood bereavement as at Sweep 8. Prevalence of childhood bereavement at Sweep 8 was determined by calculating whether each of the three categories of bereavement recorded had occurred in any of the preceding sweeps. An overarching prevalence of ‘any’ bereavement at sweep 8 was then derived, with bereaved participants defined as those with at least one ‘yes’ in any sweep against any of the bereavement categories.

An independent samples *t*-test was used to compare the mean household equivalised income at Sweep 1 of those children who had been bereaved by Sweep 8 and those children who had not. Chi-square tests were used to test for differences in tenure and DLA status at Sweep 1 between bereaved and nonbereaved children. Relative risk ratios were used to compare the overall odds of being bereaved at Sweep 8 on two dichotomous variables at Sweep 1 (lowest household equivalised income quintile *versus* highest quintile; and owned *versus* rented accommodation).

### Methodological limitations

The dataset only records whether bereavement has been experienced and does not measure the importance of these relationships to the child, the closeness of the relationship, the circumstances of the death nor the impact of the bereavement. Furthermore, multiple bereavements in one sweep (e.g. the death of both grandparents) are not captured in this dataset, nor are bereavements that fall out with the parameters of the variable (friends, neighbours, teachers or distant relatives for example). As such, it is a proxy measure of childhood bereavement.

The Significant Life Events variable was only captured from Sweep 2 onwards, and reflects events that have occurred since the previous Sweep. Thus, any bereavements that occurred between birth (or pre-birth) and Sweep 1 (conducted approximately at the age of 10 months) will not have been captured by the survey. Preliminary analyses on unweighted data (Table 1) revealed that there were significant differences between those families who had participated in all eight sweeps, and those who had dropped out of the study or missed one or more sweeps. Participants who dropped out of the study were more significantly likely to be living in rented accommodation and to have had a lower equivalised income at Sweep 1. There were no significant gender differences in dropout rates.

**Table 1.** Sample characteristics (participants *versus* dropouts, unweighted data).

	Complete data at Sweep 8 (*n* = 2815)	Incomplete data/dropout at Sweep 8 (*n* = 2402)	
Gender			
Male (*n* = 2683)	53.5%	46.5%	
Female (*n* = 2534)	54.4%	45.6%	*χ*^2^(1, *n* = 5217) = 0.387, *p* = 0.515 (ns)
Household tenure at Sweep 1			
Own (*n* = 3359)	63.4%	36.6%	
Rent (*n* = 1851)	36.9%	63.1%	*χ*^2^(1, *n* = 5210) = 337.408, *p* < 0.001***
Mean equivalised household income			
Sweep 1 (*n* = 5217)	£22,709 (SD = £12,510)	£17,570 (SD = £12,308)	*t* = 14.106, df = 4525.517, *p* < 0.001***

SD, standard deviation. *** = *p*<0.001

As discussed previously, these significant differences have important implications for this study as it is known that children living in disadvantaged circumstances are more likely to have experienced serious and multiple losses.^11^ Bereavement is also a family stressor that may increase likelihood of dropout, either in a given sweep or from the study entirely. These limitations mean that, even with the application of longitudinal weights, bereavement experiences are likely to be underrepresented in the cohort members included in this sample.

## Results

### Prevalence of childhood bereavement

**Figure 1.**
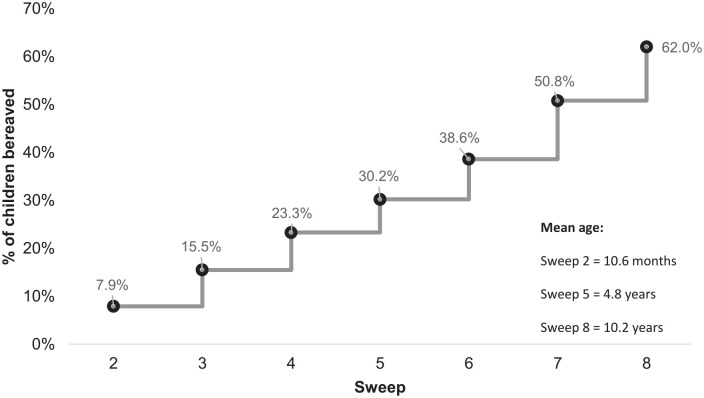
Proportion of children bereaved at each sweep (*n* = 2815).

At the time of Sweep 8, 62.0% of children had experienced the death of a grandparent or other significant relative; parent or sibling (Table 2). The death of one or more grandparents or other close relative was the most common bereavement experienced. Data indicated that by the time of Sweep 7 (mean age = 7.86, SD = 0.06), just over half of the children (50.8%) had experienced a bereavement (Figure 1). There were no significant differences in gender, with 61.4% of boys bereaved by Sweep 8, and 62.6% of girls, [*χ*^2^ (1, *n* = 2815) = 0.366, *p* = 0.545].

**Table 2.** Prevalence of childhood bereavement in Scotland at Sweep 8 (*n* = 2815, mean age = 10.18 years).

	Weighted data	
	*n*	%
Any bereavement	1744	62.0
Death of parent	38	1.3
Death of sibling	40	1.4
Death of grandparent/other close relative	1703	60.5

### Bereavement and disadvantage

The prevalence data show that bereavement becomes a majority experience before the age of 8 and analysis by SES (using the proxy measure of household equivalised income) indicates that bereavement cuts across income groups, with no significant difference in income at Sweep 1 between those who had been bereaved and those who had not (Table 3). However, these findings were accounted for by the predominance of the death of a grandparent/other close relative, which comprised the majority of bereavement experiences in the sample. Household income at Sweep 1 did not significantly differ between those children who had experienced the death of a grandparent/other close relative by the time of Sweep 8 and those who had not (Table 3). Yet, the burden for other types of bereavement did not fall equally across the sample. Household income at Sweep 1 was significantly lower for those children who had experienced the death of a parent or the death of a sibling (see Table 3).

**Table 3.** Prevalence of bereavement by household equivalised income, household tenure and Disability Living Allowance (*n* = 2815).

	Bereaved by Sweep 8		Not bereaved by Sweep 8			*p*
**Any bereavement**						
Mean equivalised income at Sweep 1	£19,389.50 (SD = £12,905)		£20,268.46 (SD = £12,345)		*t* = 1.716, df = 2548, *p* = 0.086	ns
Household tenure at Sweep 1	*n*	%	*n*	%	*χ*^2^(1, *n* = 2815) = 5.702, *p* = 0.017	*
Owned (*n* = 1787)	1077	60.3	710	39.7		
Rented (*n* = 1028)	667	64.9	361	35.1		
Disability Living Allowance at Sweep 1	*n*	%	*n*	%	*χ*^2^(1, *n* = 2808) = 4.037, *p* = 0.045	*
Claimed (*n* = 79)	58	73.4	21	26.6		
Not claimed (*n* = 2729)	1682	61.6	1047	37.3		
**Death of a grandparent or other close relative**						
Mean equivalised income at Sweep 1	£19,575.63 (SD = £12,368)		£19,951.08 (SD *=* £12,866)		*t* = 0.738, df = 2548, *p* = 0.461	ns
Household tenure at Sweep 1	*n*	%	*n*	%	*χ*^2^(1, *n* = 2814) = 1.928, *p* = 0.165	ns
Owned (*n* = 1787)	1063	59.5	724	40.5		
Rented (*n* = 1027)	639	62.2	388	37.8		
Disability Living Allowance at Sweep 1	*n*	%	*n*	%	*χ*^2^(1, *n* = 2806) = 2.190, *p* = 0.139	ns
Claimed (*n* = 78)	54	69.2	24	30.8		
Not claimed (*n* = 2728)	1644	60.3	1084	39.7		
**Death of a parent**						
Mean equivalised income at Sweep 1	£13,193.58 (SD = £11,301)		£19,813.68 (SD = £12,561)		*t* = 3.080, df = 2548, *p* = 0.002	**
Household tenure at Sweep 1	*n*	%	*n*	%	*χ*^2^(1, *n* = 2814) = 11.808, *p* = 0.0006	***
Owned (*n* = 1787)	13	0.7	1774	99.3		
Rented (*n* = 1027)	24	2.3	1003	97.7		
Disability Living Allowance at Sweep 1	*n*	%	*n*	%	*χ*^2^(1, *n* = 2807) = 11.713, *p* = 0.004	**
Claimed (*n* = 78)	5	6.4	73	93.6		
Not claimed (*n* = 2729)	33	1.2	2696	98.8		
**Death of a sibling**						
Mean equivalised income at Sweep 1	£15,652.04 (SD = £8927)		£19,769.65 (SD *=* £12,591)		*t* = 2.635, df = 34.257, *p* = 0.013	*
Household tenure at Sweep 1	*n*	%	*n*	%	*χ*^2^ (1, *n* = 2813) = 4.398, *p* = 0.036	*
Owned (*n* = 1786)	18	1.0	1768	99.0		
Rented (*n* = 1027)	21	2.0	1006	98.0		
Disability Living Allowance at Sweep 1	*n*	%	*n*	%	*χ*^2^ (1, *n* = 2806) = 28.222, *p* = 0.000	***
Claimed (*n* = 78)	7	9.0	71	91.0		
Not claimed (*n* = 2728)	32	1.2	2696	98.8		

ns, non-significant; SD, standard deviation.

* *p* < 0.05 level; **p < 0.01; ***p < 0.001.

Children whose families lived in rented accommodation at Sweep 1 were significantly more likely to have experienced bereavement overall (64.9%), as well as the death of a parent or sibling (Table 3). The proportion of those living in rented accommodation and experiencing the death of a grandparent/other close relative did not differ significantly from those who lived in owner occupied accommodation. Children who had a parent or carer claiming Disability Living Allowance at Sweep 1 were significantly more likely to have experienced a bereavement by Sweep 8 (73.4%) than their peers (61.6%), as well as the death of a parent or sibling. There was no significant difference in the prevalence of the death of a grandparent/other close relative between families with a DLA claimant and families without (Table 3).

#### Relative risk ratios

To determine the magnitude of this increased risk, unadjusted relative risk ratios were calculated (Table 4) for equivalised household income (the lowest quintile *versus* the highest quintile) and tenure type (owned *versus* rented accommodation). Analysis was not conducted for DLA due to the small number of claimants in the sample. A child born into a family in the lowest income band at Sweep 1 (less than £8410 per annum) had a five times greater risk of being bereaved of a parent by Sweep 8 than a child born into a family in the highest income band (more than £33,571 per annum). The risk of being bereaved of a sibling was 3.75 times higher in the lowest income families as it was in the highest income families, although in this instance the confidence intervals indicate that there is insufficient evidence to draw firm conclusions. Limiting the analyses on polarized income groups focuses attention on the greatest inequality, but the reduced sample size (*n* = 1024) and the low prevalence of risk in the sample (fewer than five of the children in the highest income families had been bereaved of a sibling compared to nine in the lowest income families) may have affected the results. The increased risk of experiencing the death of a grandparent/other close relative was essentially nil, and there was only a very slight increase in risk of bereavement, although for these two relative risk calculations the confidence intervals also encompass the null hypothesis value (1) and should be interpreted with caution.

**Table 4.** Relative risk ratios.

	Lowest equivalised income quintile at Sweep 1) (<£8410)		Highest equivalised income quintile at Sweep 1) (>£33,571)		Relative risk ratio	95% confidence intervals	
	*n*	%	*n*	%		Lower	Upper
Bereaved of a parent	18	3.2	-	-	5.022	1.488	16.942
Bereaved of a sibling	9	1.6	-	-	3.75	0.814	17.270
Bereaved of a grandparent/other relative	332	59.5	276	59.1	1.007	0.909	1.115
Any bereavement	352	63.1	276	59.2	1.065	0.965	1.175
	Rented accommodation at Sweep 1		Owner occupied accommodation at Sweep 1		Relative risk ratio	95% confidence intervals	
	*n*	%	*n*	%		Lower	Upper
Bereaved of a parent	24	2.3	13	0.7	3.212	1.643	6.281
Bereaved of a sibling	21	2.0	18	1.0	2.029	1.086	3.790
Bereaved of a grandparent/other relative	639	62.2	1063	59.5	1.046	0.984	1.112
Any bereavement	667	64.9	1077	60.3	1.077	1.015	1.142

While household tenure type was a crude and proxy measure for SES, analysis indicated that the variable did measure disadvantage that put children at increased risk of bereavement. For three of the bereavement types (death of a parent, sibling and overall bereavement), there was an increased risk of bereavement for children living in rented or other accommodation at Sweep 1, compared to those children living in owner occupied accommodation (Table 4). For a child living in rented or other accommodation, the risk of a parent dying was three times greater, the risk of a sibling dying was approximately two times more likely but the increased risk of experiencing any bereavement overall was essentially nil, at just over one (equating to a 7.7% increase in risk).

## Discussion

This article is the first published study of the prevalence of childhood bereavement in Scotland, drawing upon a large, nationally representative sample and collating children’s bereavement experiences from 10 months until approximately 10 years. It is also the first large-scale prevalence study undertaken in the United Kingdom for more than 15 years and, unlike other published studies,^2,45^ it draws upon detailed longitudinal data in which the timing and extent of bereavement can be ascertained. The findings presented here therefore represent an important touchstone for policymakers, practitioners, academics and wider civil society.

The findings reveal that by the age of 8, more children will have experienced the death of a close family member (50.8%) than those who have not, and that almost two-thirds of children (62.0%) will have experienced a close family bereavement by the age of 10. Yet, the study limitations outlined in the methodology, including a narrow focus on bereavement experiences (limited to a parent or carer, sibling or grandparent/close relative); no available data about bereavements that occurred before the age of 10 months; bereavement being a probable cause for some families to drop out of the study; an inability to measure multiple bereavements of the same type within an individual sweep (i.e. the death of two grandparents) and a data cut-off at a mean age of 10 years and 2 months, mean that the findings presented here are, without a doubt, an underestimate of the true extent of childhood bereavement in Scotland. Furthermore, the increase in the rate of exposure to bereavement throughout each of the sweeps in this longitudinal study signifies the potential for an overall prevalence of childhood bereavement (up until the age of 18) that far outstrips any previous estimates of childhood bereavement based on surveys with smaller school-based samples of adolescents.^2,8^ A number of reasons may account for this, particularly in relation to study design: in the GUS study, data are collected timeously (rather than retrospectively) from a nationally representative sample of children which encompasses those who may not be engaged in school or participate in school surveys.

The high and widespread prevalence of bereavement indicates that children’s bereavement needs are not being, and cannot be met, by professional services alone, indeed, as Breen and colleagues^9^ argue, such an approach would be ‘neither effective nor economical’. It suggests that most children cope with bereavement and that there is a need to better understand and learn from these experiences to support what Aoun^32^ calls ‘everyday’ assets in the community, ‘without over-reach from professional services’ (p. 6). This supports the need to reframe bereavement as a public health issue, whereby focus is given to looking beyond individual experiences to the social conditions with which children become bereaved and the resources and deficits within communities that support or inhibit these experiences. Current bereavement care predominantly attends to presenting issues; adopting public health approaches invites a range of stakeholders, including service providers, education, community groups and lay people to consider bereavement issues before they are presented.^46^ As such, the relevance of a tiered model of support that recognizes and promotes the capacity of informal care networks as an equal partner in bereavement support is highlighted. For example, this may include developing death and grief literacy in children by adopting universal, age-appropriate, curriculum attention within early years and primary education; developing bereavement affirming policies and practices in the social spaces where children inhabit; and ensuring that all people (family members and professionals) involved with children feel ready to engage with them about these issues. This potentially requires significant culture change in society about the willingness and ability to have open and honest conversations with children, as well as implications for professional training and children’s services, including those offering specialist bereavement support such as palliative care services.

The findings indicate that bereavement in childhood tends to be limited to what might be seen as more ‘normative’ bereavement experiences, such as the death of a grandparent. This identifies the relevance of low-level interventions which focus on normalizing bereavement and grief with children and further supports the need for a tiered approach to bereavement.^38^ Such intervention may require developing and supporting the capacity of families, peers and community networks to facilitate such an approach, what Breen and colleagues^9^ view as supporting the resilience of communities. However, the findings also indicate that children who live in families that may already be at a disadvantage (by income, tenure or health status) are at increased risk of experiencing the death of a parent or sibling. While each child is an individual, and each bereavement will be experienced differently, experiencing the death of a household member, or someone who has a caring role, may have a long-term or more severe impact than, for example, the death of an elderly grandparent who resides outside of the household. For children who are already more vulnerable, targeted policies and specialist support may be required to ensure that they receive timely and appropriate support and do not fall through cracks in the system. Furthermore, experiencing a bereavement may add to family stress, instability, inequality and disadvantage, with those who are already vulnerable shouldering the majority of the bereavement burden. Thus, identifying vulnerable groups and working together to explore and develop meaningful support networks and services is essential. Situating childhood bereavement within public health frameworks provides an opportunity to incorporate these experiences within a wider range of action and perspectives that aim to reduce social and health inequalities that are related to bereavement. While the relationship between childhood bereavement and public health requires further theoretical and practical engagement,^47^ it suggests a reorientation of current bereavement service delivery to one that recognizes and promotes the role of community, educational and social networks.

There are some areas that are worthy of further exploration, and in which more research is required. One obvious limitation of this study is that it only documents bereavements experienced by the age of 10 and long-term analysis using later sweeps of data will be essential. Unpicking the nature of the interaction between bereavement and disadvantage is complex, and was out of scope of this study, in which the overarching aim was to formally document the prevalence of childhood bereavement. However, increasing health and mortality inequalities^48^ are likely to be an important factor, and monitoring the long-term impact of bereavement on families and children in relation to family stress, disruption and disadvantage will also be important for both fully understanding children’s bereavement experiences and improving outcomes.

Of note is that, in this study, responses about bereavement were completed by parents, whereas in other studies that estimate the prevalence of childhood bereavement, the responses tend to come directly from young people themselves.^2,8^ This is important given that it is widely recognized that it is the meaning that children give to bereavement that is key to how they understand and cope with their experience.^20,49,50^ While this methodological approach was, in part, necessitated by the age of the children participating in the early sweeps of the GUS research, it means that this study does not account for children’s relational experiences of bereavement but instead parent/carer recollection. The omission of children’s views is important given that a public health approach to childhood bereavement would place emphasis on keeping their views central to the process of contextualizing bereavement experiences and shaping support. Furthermore, more research is needed on how children who do not use specialist support adapt and cope with bereavement and what can be learnt from these experiences. Such research will have implications for understanding children’s experiences of loss more broadly.

To summarize, the findings identify that children are not protected from death by virtue of their age and that the extent of childhood bereavement in the sample means that it is not feasible, nor desirable, for children experiencing bereavement to be supported by professionals alone. Reframing childhood bereavement as a public health issue presents an opportunity for a significant change in childhood bereavement care by placing emphasis on a multifaceted approach that firmly situates children within the families and communities in which they live to better understand and respond to bereavement needs.

## References

[1] Haine RA Ayers TS Sandler IN, et al Evidence-based practices for parentally bereaved children and their families. Prof Psychol Res Pr 2008; 39: 113–121. 20585468 10.1037/0735-7028.39.2.113 PMC2888143

[2] Harrison L Harrington R. Adolescents’ bereavement experiences: prevalence, association with depressive symptoms and use of services. J Adolesc 2001; 24: 159–169. 11437477 10.1006/jado.2001.0379

[3] Spuij M Reitz E Prinzie P, et al Distinctiveness of symptoms of prolonged grief, depression, and post-traumatic stress in bereaved children and adolescents. Eur Child Adolesc Psychiatry 2012; 21: 673–679. DOI: 10.1007/s00787-012-0307-4. 22791348 PMC3506830

[4] Dillen L Fontaine J Verhofstadt-Denève L. Confirming the distinctiveness of complicated grief from depression and anxiety amongst adolescents. Death Stud 2009; 33: 437–461. 19469074 10.1080/07481180902805673

[5] Parsons S. Long-term impact of childhood bereavement: preliminary analysis of the 1970 British Cohort Study (BCS70), 2011, https://www.gov.uk/government/publications/long-term-impact-of-childhood-bereavement-preliminary-analysis-of-the-1970-british-cohort-study-bcs70

[6] Vaswani N. The ripples of death: exploring the bereavement experiences and mental health of young men in custody. Howard J Crim Justice 2014; 53: 341–359.

[7] Abdelnoor A Hollins S. The effect of childhood bereavement on secondary school performance. Educ Psychol Pract 2004; 20: 43–54. DOI: 10.1080/0266736042000180401.

[8] Del Carpio L Rasmussen S Paul S A theory-based longitudinal investigation examining predictors of self-harm in adolescents with and without bereavement experiences. Front Psychol 2020; 11: 1153 DOI: 10.3389/fpsyg.2020.01153. PMC7283530 32581958

[9] Breen LJ Aoun SM O’Connor M, et al Bridging the gaps in palliative care bereavement support: an international perspective. Death Stud 2014; 38: 54–61. DOI: 10.1080/07481187.2012.725451. 24521046

[10] De E. A Literature Review into the Prevalence and Impact of Loss and Bereavement on Individuals Experiencing Severe and Multiple Disadvantage. London: Lankelly Chase Foundation, 2018.

[11] Ribbens McCarthy J Jessop J Young People, Bereavement and Loss: Disruptive Transitions? London: National Children’s Bureau, 2005.

[12] Cerel J Fristad MA Verducci J, et al Childhood bereavement: psychopathology in the two years postparental death. J Am Acad Child Psy 2006; 45: 681–690. DOI: 10.1097/01.chi.0000215327.58799.05. 16721318

[13] Worden W. Grief and Children: When a Parent Dies. New York: Guildford Press, 1996.

[14] Brewer JD Sparkes AC. Young people living with parental bereavement: insights from an ethnographic study of a UK childhood bereavement service. Soc Sci Med 2011; 72: 283–290. 21146275 10.1016/j.socscimed.2010.10.032

[15] Paul S. Is death taboo for children? Developing death ambivalence as a theoretical framework to understand children’s relationship with death, dying and bereavement. Child Soc 2019; 33: 556–571. DOI: 10.1111/chso.12352.

[16] Currier JM Holland JM Neimeyer RA. The effectiveness of bereavement interventions with children: a meta-analytic review of controlled outcome research. J Clin Child Adolesc Psychol 2007; 36: 253–259. DOI: 10.1080/15374410701279669. 17484697

[17] Bergman AS Axberg U Hanson E. When a parent dies – a systematic review of the effects of support programs for parentally bereaved children and their caregivers. BMC Palliat Care 2017; 16: 39 DOI: 10.1186/s12904-017-0223-y. PMC5553589 28797262

[18] Akerman R Statham J. Bereavement in Childhood: The Impact on Psychological and Educational Outcomes and the Effectiveness of Support Services. London: Childhood Wellbeing Research Centre, 2014.

[19] Scott R Wallace R Audsley A, et al Young people and their understanding of loss and bereavement. Bereavement Care 2019; 38: 6–12. DOI: 10.1080/02682621.2019.1588560.

[20] Ribbens McCarthy J Young People’s Experiences of Loss and Bereavement: Towards and Interdisciplinary Approach. Maidenhead; New York: Open University Press, 2006.

[21] Raveis V Siegel K Karus V. Children’s psychological distress following the death of a parent. J Youth Adolescence 1999; 28: 165–180.

[22] Ellis J Dowrick C Lloyd-Williams M. The long-term impact of early parental death: lessons from a narrative study. J R Soc Med 2013; 106: 57–67. DOI: 10.1177/0141076812472623. PMC3569022 23392851

[23] Jamieson L Highet G. Troubling loss: children’s experiences of major disruptions in family life. In: Ribbens McCarthy J Hooper C-A Gillies V (eds) Family Troubles? Exploring Changes and Challenges in the Family Lives of Children and Young People. Bristol: Policy Press, 2013, pp. 135–150.

[24] Cross S. ‘I can’t stop feeling sad’: Calls to ChildLine About Bereavement. London: ChildLine, 2002.

[25] Servaty H Hayslip B. Adjustment to loss among adolescents. Omega 2001; 43: 311–330.

[26] Silverman PR Worden JW. Children’s reactions in the early months after the death of a parent. J Orthopsych 1992; 62: 93–104. 10.1037/h0079304 1546765

[27] Stjernswärd J Foley KM Ferris FD. The public health strategy for palliative care. J Pain Symptom Manag 2007; 33: 486–493. DOI: 10.1016/j.jpainsymman.2007.02.016. 17482035

[28] Kellehear A. Health-promoting palliative care: developing a social model for practice. Mortality 1999; 4: 75–82.

[29] Conway S. The changing face of death: implications for public health. Crit Public Health 2007; 17: 195–202.

[30] Aoun SM Breen LJ White I, et al What sources of bereavement support are perceived helpful by bereaved people and why? Empirical evidence for the compassionate communities approach. Palliat Med 2018; 32: 1378–1388. DOI: 10.1177/0269216318774995. 29754514 PMC6088515

[31] Aoun SM Breen LJ Rumbold B, et al Matching response to need: what makes social networks fit for providing bereavement support? PLoS ONE 2019; 14: e0213367. DOI: 10.1371/journal.pone.0213367. PMC6405096 30845193

[32] Aoun SM. Bereavement support: from the poor cousin of palliative care to a core asset of compassionate communities. Progress Palliat Care 2020; 28: 107–114.

[33] Rumbold B Aoun SM. Bereavement and palliative care: a public health perspective. Progress Palliat Care 2014; 22: 131–135.

[34] Baum F. The New Public Health. Melbourne, VIC, Australia; New York: Oxford University Press, 2008.

[35] Aoun SM Breen LJ O’Connor M, et al A public health approach to bereavement support services in palliative care. Aust NZ J Publ Heal 2012; 36: 14–16. DOI: 10.1111/j.1753-6405.2012.00825.x. 22313700

[36] Petersen AR Lupton D. The New Public Health: Health and Self in the Age of Risk. London: SAGE, 1997.

[37] Moran-Ellis J. Reflections on the sociology of childhood in the UK. Curr Sociol 2010; 58: 186–205. DOI: 10.1177/0011392109354241.

[38] Jones A Deane C Keegan O. The development of a framework to support bereaved children and young people: the Irish Childhood Bereavement Care Pyramid. Bereavement Care 2015; 34: 43–51.

[39] Dempers C Gott M. Which public health approach to palliative care? An integrative literature review. Progress Palliat Care 2016; 25: 1–10. DOI: 10.1080/09699260.2016.1189483.

[40] Government S. Public Health Priorities for Scotland. Edinburgh: Scottish Government, 2018.

[41] ScotCen. Growing Up in Scotland: survey design and methodology: sampling, response and weighting, 2014, https://growingupinscotland.org.uk/wp-content/uploads/2014/03/Data-Wshop-2014_Intro-to-data1_survey-design.pdf

[42] ScotCen. Growing Up in Scotland Sweep 8 – 2014/15 User Guide, n.d., https://growingupinscotland.org.uk/wp-content/uploads/2017/11/GUS-BC1-SW8-User-Guide.pdf

[43] Government U. Disability Living Allowance (DLA) for adults, n.d., https://www.nidirect.gov.uk/articles/disability-living-allowance-adults#:~:text=Disability%20Living%20Allowance%20(DLA)%20is,16%20and%20State%20Pension%20age

[44] Corp. I. IBM SPSS Statistics for Windows, Version 25.0. Armonk, NY: IBM Corp, 2017.

[45] Fauth B Thompson M Penny A. Associations Between Childhood Bereavement and Children’s Background, Experiences and Outcomes: Secondary Analysis of the 2004 Mental Health of Children and Young People in Great Britain Data. London: National Children’s Bureau, 2009.

[46] Paul S. Advancing Education and Support Around Death, Dying and Bereavement: Hospices, Schools and Health Promoting Palliative Care. Edinburgh: University of Edinburgh, 2015.

[47] Whitelaw S Clark D. Palliative care and public health: an asymmetrical relationship? Palliat Care 2019; 12: 1–14. DOI: 10.1177/1178224218819745. PMC6383085 30814842

[48] McCartney G Popham F Katikireddi S, et al How do trends in mortality inequalities by deprivation and education in Scotland and England & Wales compare? A repeat cross-sectional study. BMJ Open 2017; 7: e017590. DOI: 10.1136/bmjopen-2017-017590. PMC5642664 28733304

[49] Davies H. Embodied and sensory encounters: death, bereavement and remembering in children’s family and personal lives. Child Geogr 2017; 17: 552–564.

[50] Evans R. Parental death as a vital conjuncture? Intergenerational care and responsibility following bereavement in Senegal. Soc Cult Geogr 2014; 15: 547–570. DOI: 10.1080/14649365.2014.908234.

